# Co-occurrence of Malignant Melanoma and Squamous Cell Carcinoma on Burn Scars: A Case Report

**DOI:** 10.7759/cureus.44283

**Published:** 2023-08-28

**Authors:** Naoki Sasaki, Natsuko Saito-Sasaki, Sanehito Haruyama, Yu Sawada

**Affiliations:** 1 Dermatology, University of Occupational and Environmental Health, Kitakyushu, JPN; 2 Dermatology, Haruyama Dermatology Clinic, Gyota, JPN

**Keywords:** co-existence of skin cancers, literature review, case report, squamous cell carcinoma, malignant melanoma

## Abstract

Tumors arising from burn scars are not rare but sometimes cause the rare co-existence of different tumors. However, detailed information on this topic remains largely unknown. We present a case of the co-occurrence of malignant melanoma and squamous cell carcinoma in a patient with a history of burn scars. A 73-year-old man presented with an erythematous plaque on his left lower leg that gradually turned into a tumor with ulceration. He also presented with scaly tumors at other sites within the same burn scar lesion. He had a history of burns on the left leg at the age of 20 years. After surgical resection of the tumors, histological analysis revealed that the posterior aspect of the largest tumor was malignant melanoma, and the remaining two tumors were squamous cell carcinomas, indicating the co-existence of different types of malignant skin cancers. Based on a literature review of previously published case reports, this is the first report to highlight the importance of complete skin grafts in reducing this risk.

## Introduction

The etiologies of cutaneous malignant tumors are multifactorial and coexist with other types of skin cancer in the same individuals. Because the triggers of malignant tumors sometimes drive common key oncogenesis enhancers [1-3], it has been speculated that the same causal forces of malignant tumors might be the cause of other malignant tumors in the skin. Here, we present the co-occurrence of malignant melanoma and squamous cell carcinoma in a patient with a history of burn scars.

## Case presentation

**Figure 1:**
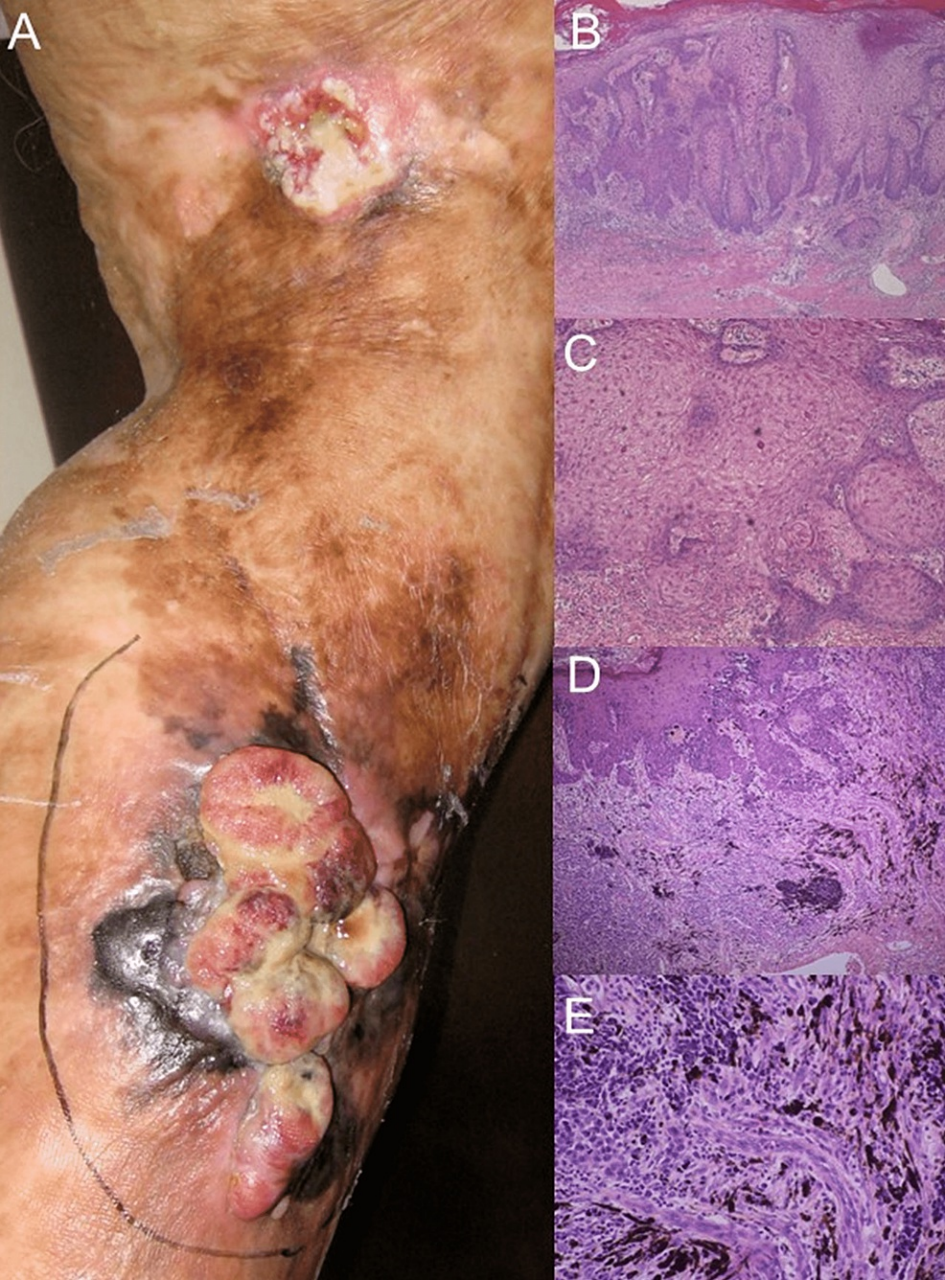
Clinical manifestation and histological analysis (A) Clinical characteristics burn scars are overlaid throughout his left lower thigh, and yellowish necrotic tissue covers an erythematous tumor with ulceration surrounding black macules located on his left lower leg (malignant melanoma). Erythematous tumors with yellow necrotic tissue on the surface are present on the left popliteal fossa with a 4 mm erythematous plaque and a 3 cm tumor (squamous cell carcinoma). (B, C) Histological examination of the squamous cell carcinoma at low magnification (B) and high magnification (C). (D, E) Histological examination of malignant melanoma in low-magnification (D) and high-magnification (E) views.

A 73-year-old man presented with an erythematous plaque on his left lower leg that gradually turned into a tumor with ulceration. He also presented with scaly tumors at other sites within the same burn scar lesion. He had a history of burns on the left leg at the age of 20 years. The patient underwent skin grafting twice, and the left skin wound healed spontaneously. Physical examination revealed burn scars throughout his left lower thigh and yellowish necrotic tissue covering an erythematous tumor with ulceration surrounding the black macules located on his left lower leg. The tumor measured 15 cm × 10 cm (Figure 1A). In addition, erythematous tumors with yellow necrotic tissue on the surface were present on the left popliteal fossa with a 4 mm erythematous plaque and a 3 cm tumor (Figure 1A). After wide surgical resection of these tumors, histological analysis revealed that the posterior aspect of the largest leg tumor was malignant melanoma, and the remaining two tumors were squamous cell carcinomas (Figure 1B-E). This implied the co-existence of different types of malignant skin cancers. Additional surgical resection margins and sentinel lymph node biopsies were performed. Computed tomography and histological examinations of the lymph node biopsy specimen showed no distant or lymph node metastases. He has had no recurrence or metastasis of the tumor for 5 years.

## Discussion

Burn scars are essential risk factors for the development of malignant tumors. A previous study of 412 instances of malignant tumors developing from burn scars revealed that squamous cell carcinoma is the most prevalent skin cancer. In addition, malignant melanoma accounts for approximately 6% of cases of burn scar-associated cutaneous malignancies [4]. Since we present a rare case of co-occurrence of malignant melanoma and squamous cell carcinoma, careful observation is required to monitor the recurrence of the tumors. We searched Pubmed for similar cases and six cases of malignant melanoma and squamous cell carcinoma on burn scars were described in detail in English literature (Table 1) [2,5-8].

**Table 1:** The summary of the co-existence of melanoma and squamous cell carcinoma following burn scars

Author	Sex	The onset age of tumor	Co-existence	History of burn	Outcome	Treatment
Muhlemann et al. [5]	Male	59	Same site	Unknown (Spontaneous healing)	Unknown	Surgical resection
Walker et al. [2]	Female	78	Different sites	11 years old (spontaneous healing)	Unknown	Surgical resection
Akiyama et al. [6]	Male	55	Same site	4 years old (skin graft + spontaneous healing)	Survival 9 years after surgery	Surgical resection, plus lymph node dissection
Ikeda et al. [7}	Male	47	Different sites	4 years old (spontaneous healing), surgical resection plus skin graft for SCC 2 years later arising melanoma	Survival 16 months after surgery	Surgical resection, plus lymph node dissection, plus DAV-feron therapy
Alcochel et al. [8]	Female	46	Different sites	6 years old (spontaneous healing), surgical resection, plus skin graft for malignant fibrous histiocytoma (37 years old)	Dead 15 months after the surgery	Surgical resection lymph node dissection TP-1 treatment
Our case	Male	73	Different sites	20 years old (skin graft + spontaneous healing)	Alive 5 years after the surgery	Surgical resection Lymph node biopsy

The average duration of occurrence of malignant melanoma and squamous cell carcinoma was 50.8 years. Four patients (66.7%) had different sites of melanoma and squamous cell co-occurrence. Although there was an insufficient observation period for these cases, only one patient died after treatment. All patients exhibited burn scars with spontaneous healing.

## Conclusions

We report a rare case of the co-existence of malignant melanoma and squamous cell carcinoma in previous burn scars. Therefore, dermatologists should consider the possibility of the co-existence of different skin cancers on burn scars arising from cutaneous tumors before surgical resection. In addition, complete skin graft surgery may be essential to reduce the risk of co-occurrence of melanoma and squamous cell carcinomas. To the best of our knowledge, this is the first report to highlight the importance of complete skin grafts in reducing this risk.
